# Selected Papers from the 4th International Conference on Bioinspired Systems and Cognitive Signal Processing

**DOI:** 10.1155/2011/913040

**Published:** 2011-10-31

**Authors:** Fabio Babiloni, Andrzej Cichocki, Saeid Sanei, Laura Astolfi, Febo Cincotti, Sara Gonzalez Andino

**Affiliations:** ^1^Department of Physiology and Pharmacology, Sapienza University of Rome, 00185 Rome, Italy; ^2^Laboratory for Advanced Brain Signal Processing Riken, Brain Science Institute, Wako, Saitama 351-0198, Japan; ^3^Faculty of Engineering and Physical Sciences, University of Surrey, Guildford Surrey GU2 7XH, UK; ^4^Department of Computer and System Sciences, Sapienza University of Rome, 00185 Rome, Italy; ^5^Neuroelectrical Imaging and BCI Lab, Fondazione Santa Lucia Via Ardeatina, 306 I-00179 Rome, Italy; ^6^Electrical Neuroimaging Group, Department of Clinical Neurosciences, Faculty of Medicine, University Hospital of Geneva, University of Geneva, Geneva, Switzerland

## 1. Introduction

This special issue is devoted to ‘‘Selected Papers from the 4th International Conference on Bioinspired Systems and Cognitive Signal Processing.” This peer-reviewed special issue is one product of the International Conference on Bioinspired systems and Signal Processing (Biosignals 2011), as part of the International Joint Conference on Biomedical Engineering Systems and Technologies, Biostec (http://www.biosignals.biostec.org/BIOSIGNALS2011/home.asp), which was held in Rome, during 26–29 January 2011. This issue was promoted by the IEEE Technical Committee of Biomedical Signal Processing (IEE TC-BSP).

The goal of this special issue is to convey mainly several messages which arose from this prestigious and intense conference. The major focus in this meeting was biomedical signal processing, where several areas of interest to the researchers in this field were comprehensively covered. This issue is therefore to report some highlighted contributions by a number of prominent researchers in the related field. Interested readers will find in this special issue papers that belong broadly to the areas of neuroscience, cardiovascular signals, behaviour, body movement, and modelling. These areas have been spanned by a number of articles in computational modelling and intelligence, neuroimaging, neuromarketing, body movement and gait analysis, brain-computer interfacing, electrocardiography and analysis of cardiovascular signals, neurocomputing and retinal image matching technique.

What follows is a brief editorial review of the topics covered by the published papers in this special issue.

## 2. Neuroscience

This area of science has received much attention in recent decades, and tremendous contributions from a simple model to more complicated and powerful models to describe the behaviour of neurons, cerebral networks [1] up to the description of spatiotemporal EEG distributions [2] have been made by the workers in this field. This special issue starts with a paper dealing with the analysis of the properties of spiking neurons in order to capture some essence of the concept of “consciousness” by M. Ebner and S. Hameroff, 2011 and will continue with the analysis of very large spatial electromagnetic fields in connection with high resolution EEG technologies by G. Vecchiato et al., 2011. In particular the paper of Ebner and Hameroff describes the implementation of a single layer neural network to perceive and represent a visual scene. The implemented system demonstrates a moving zone of synchrony which correlates with figure ground separation as proposed in the conscious pilot model presumedly responsible for converting nonconscious “autopilot” cognition to consciousness. By moving from the modelling of neurons spiking to the analysis of scalp potential distributions, this special issue presents a couple of contributions in the area of Brain Computer Interface. Such area of science investigate the possibility of the voluntary modulation of brain responses recorded over the scalp and their use for machine learning and human-computer handshaking. This attempt often requires powerful classifiers to discriminate between the features used for making decisions. It is therefore crucial for such classifiers to have high performance and to be optimised for each particular application. In this respect, the contribution of D. Devlaminck et al., 2011 is directed toward an improvement of a particular classifier commonly used in BCI field. They reduced the amount of calibration data that is needed for the proper classification of a new subject, by applying a multitask machine learning technique to preprocessing the phase. Comparisons of BCI classifiers are instead the argument for the contribution of N. V. Manyakov et al., 2011, who have focused on typing accuracy of a speller device to be used with BCI given the individual patient's disorder, and how it correlates with the type of classifier used. In this paper, they also discuss a number of recommendations to be considered when building a P300-based typing system for disabled subjects. 

Next we move from BCI to the use of high resolution EEG to capture the information regarding neuroimaging and its application in neuromarketing. Application of the today neuroscience techniques to marketing can be of help in establishing a physiologically inspired assessment criterion for many areas of marketing. Neuromarketing and its development within the community is another interesting new topic explored in the work by G. Vecchiato et al., 2011. Particularly, the correlations between different properties of the neurophysiological cerebral signals and the nature of the advertising stimuli have been addressed. As an example, the correlation between the steady state visually evoked potential latency and long-term memory, when observing a commercial video, has been studied. From these studies it has been anticipated that electrophysiological signs may be used as a probe to find out how pleasant a commercial stimulus is. A number of applications related to neuromarketing have also been discussed regarding the cortical activity influenced by the advert effect. Characteristics of a video product, for instance, can be tuned by assessing the information achieved using brain imaging techniques. As the conclusion of this work, neuroimaging, particularly EEG, can open its space in social networking with special effect on marketing.

## 3. The Use of Cardiovascular Signals and Images

The selected contributions for this area of the special issue are related to the application of DSP and pattern recognition tools and algorithms to heart and vascular signals for various purposes. The first paper is related to the use of unsupervised and interpatient classification of heart beats often used as the starting point in many applications where long-term monitoring of the cardiac functions is required. As in any classification problem, there is an inherent tradeoff between the efficiency of classification and the number of features that have to be used in the classification. In the work of G. Doquire et al., 2011 some feature selection techniques have been considered to extract optimal feature subsets for the state-of-the-art ECG classification models. The performances have been evaluated using real ambulatory recordings and compared to previously reported feature choices using the same models. The results indicate that a small number of individual features actually serve the classification and that better performances can be achieved by removing unnecessary features. In the second application A. Lourenço et al., 2011 propose a finger based ECG biometric system that uses signals collected from the fingers, through a minimally intrusive 1-lead ECG setup recurring to Ag/AgCl electrodes without gel as interface with the skin. The collected signal is significantly noisier than the ECG acquired from around the chest sites, motivating the application of feature extraction and signal processing techniques to the problem. Again with the possible application in the field of biometry, the third application provided in this area by A. Bhuiyan et al., 2011 is related to a method for the retinal image matching that can be used in human recognition and identification or the longitudinal study of patients. Vascular invariant features are extracted from the retinal image, and a feature vector is constructed for each of the vessel segments in the retinal blood vessels. The feature vectors are represented in a tree structure with maintaining the vessel segments actual hierarchical positions. Using these feature vectors, the corresponding images are cross-matched. The method identifies the same vessel in the corresponding images for comparing the desired feature(s). Initial results are encouraging and demonstrate that the proposed method is suitable for image matching and patient longitudinal study.

## 4. Behavior, Body Movement, and Modeling

In this part of the special issue different methodologies have been applied to the study of different aspects of the behavior. Starting from the overt actual behavior, a paper of T. Watanabe et al., 2011 attacks the problem by developing a wearable sensor system for gait evaluation using gyroscopes and accelerometers. The outcome is applied to rehabilitation, healthcare, and so forth. Simultaneous measurements from the joint angles of the lower limbs and stride length were tested in the detection process using a prototype of wearable sensor system. Their results suggested that the wireless wearable inertial sensor system could detect characteristics of gait, and this result could be interesting in the rehabilitation framework. The paper provided by A. Mannini and A. M. Sabatini, 2011 is related to the processing of body movement data. In particular, while the previous paper is related to the development of particular sensors, this paper is instead concerned with the data processing obtained from accelerometers. In fact, it is important to understand from such kind of data the behavioral characteristic of the person, since automatic classification of human physical activities is highly attractive for pervasive computing systems, whereas contextual awareness may ease the human-machine interaction, while in biomedicine, wearable sensor systems are proposed for long-term monitoring. The paper is concerned with the machine learning algorithms needed to perform the classification task. Hidden Markov Model (HMM) classifiers are studied by contrasting them with Gaussian Mixture Model (GMM) classifiers. A different approach is proposed by T. Hinze et al., 2011 in which the question of whether biological control systems for regulation of oscillatory signals and their technical counterparts utilize similar mechanisms has been dealt with. 

It has been concluded that if the answer to the above question is positive, the modeling approaches and parameterization adopted from the building blocks can help to identify the general components for frequency control in circadian clocks along with gaining insight into mechanisms of clock synchronization to external stimuli like the daily rhythm of sunlight and darkness. As a first step in this direction, the authors demonstrated that a model of coupled repressilators is able to synchronise the clock signals in a monofrequential manner. 
